# Insights into autophagosome biogenesis from *in vitro* reconstitutions

**DOI:** 10.1016/j.jsb.2016.04.005

**Published:** 2016-10

**Authors:** Eleonora Turco, Sascha Martens

**Affiliations:** Max F. Perutz Laboratories, University of Vienna, Vienna Biocenter, Dr. Bohr-Gasse 9, 1030 Vienna, Austria

**Keywords:** Membrane, Autophagy, Autophagosome, *In vitro* reconstitution, Organelle biogenesis

## Abstract

Macro-autophagy (autophagy) is a conserved catabolic pathway for the degradation of cytoplasmic material in the lysosomal system. This is achieved by the sequestration of the cytoplasmic cargo material within double membrane-bound vesicles that fuse with lysosomes, wherein the vesicle’s inner membrane and the cargo are degraded. Autophagosomes form in a *de novo* manner and their precursors are initially detected as small membrane structures that are referred to as isolation membranes. The isolation membranes gradually expand and subsequently close to give rise to autophagosomes. Many proteins required to form autophagosomes have been identified but how they act mechanistically is still enigmatic. Here we critically review reconstitution approaches employed to decipher the inner working of the fascinating autophagy machinery.

## 1 Introduction

Macroautophagy (hereafter autophagy) is a degradative pathway that contributes to cell homeostasis, both in normal and under stress conditions. Initially, autophagy was considered a non-selective degradation pathway in which a random portion of cytoplasm is degraded and recycled in response to cellular stress. However, it is now established that autophagy can be a highly selective process that serves to specifically degrade protein aggregates, organelles (mitochondria, peroxisomes, endoplasmic reticulum) and pathogens (Reggiori et al., 2012). Basal levels of selective autophagy are independent of external stimuli like starvation or oxidative stress and are of primary importance in maintaining cell homeostasis (Mizushima et al., 2008, Zaffagnini and Martens, 2016).

**Fig. 1:**
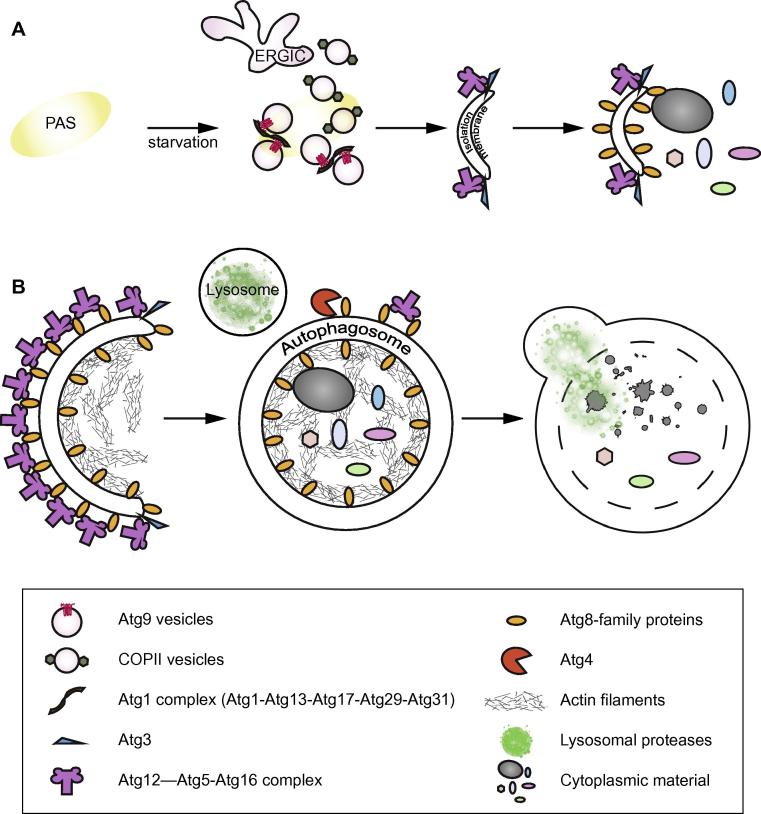
Schematic representation of autophagy. (A) Upon starvation the Atg1 kinase complex is recruited to the PAS, where it tethers and organizes Atg9 vesicles. ERGIC derived COPII vesicles, competent in Atg8-family protein lipidation are also recruited to the PAS. Subsequently, an isolation membrane is generated and decorated with Atg8-family proteins. (B) The isolation membrane expands to enwrap cytoplasmic cargo material while different mechanisms are at work to maintain its shape. Later, the isolation membrane closes, generating a mature autophagosome, which in turn fuses with the lysosome leading to the degradation of the inner autophagosomal membrane and the cytoplasmic cargo material.

Autophagy entails the *de novo* formation of a double membrane bound organelle: the autophagosome, which enwraps cytoplasmic material and eventually fuses with the lysosome/vacuole, resulting in the degradation of its cargo by lysosomal hydrolases (Fig. 1).

Forty-one autophagy related genes (ATGs), involved in autophagosome formation, were identified in yeast and many of them are conserved in higher eukaryotes. A subset of these genes referred to as the autophagic core machinery is required for selective and non-selective types of autophagy (Suzuki and Ohsumi, 2007, Yang and Klionsky, 2010). Upon induction of autophagy by starvation or by the presence of cargo, autophagosome formation in yeast is initiated at a perivacuolar site named pre-autophagosomal structure (PAS) (Suzuki and Ohsumi, 2010, Suzuki et al., 2001). In mammalian cells multiple isolation membrane assembly sites are formed upon autophagy induction (Itakura and Mizushima, 2010). A coordinated recruitment of ATG proteins (Suzuki et al., 2007) and other factors to the PAS leads to the formation of a cup shaped isolation membrane (or phagophore), which elongates and finally closes to form a mature autophagosome (Fig. 1). The *S. cerevisiae* Atg1 kinase forms a complex with Atg13, Atg17, Atg29 and Atg31 and it is necessary for autophagy initiation at the PAS (Stjepanovic et al., 2014, Suzuki et al., 2007). The complex is largely conserved in mammalian cells, where the kinase ULK1 acts in complex with ATG13, FIP200 and ATG101 to activate autophagy (Mizushima, 2010). Atg17 acts as a scaffold that recruits and organizes Atg9 vesicles at the PAS (Sekito et al., 2009) (Fig. 1A). Atg9 (mATG9 in mammals) is the only transmembrane protein among the ATGs and it is necessary for autophagosome formation (Lang et al., 2000, Noda et al., 2000).

Downstream of the Atg1 complex the phosphatidylinositol-3 kinase (PI3K) complex is recruited to the PAS (Suzuki et al., 2007), where it mediates the conversion of phosphatidylinositol (PI) into phosphatidylinositol-3 phosphate (PI3P) (O Farrell et al., 2013). The PI3K complex is composed of the catalytic subunit Vps34 (PI3KC3 in mammals), Atg6 (Beclin 1), Vps15 (p150) and Atg14 (ATG14L/Barkor). It is not entirely clear how the complex is recruited to the PAS, but it was suggested that the subunit Atg14 is targeted to autophagic membranes (Matsunaga et al., 2010). The HORMA domain of Atg13 might also have a role in the indirect recruitment of PI3K to the PAS (Jao et al., 2013, Suzuki et al., 2015b). Accumulation of PI3P on the isolation membrane acts as a platform for the recruitment of WIPIs/PROPPINs (Baskaran et al., 2012, Krick et al., 2012, Suzuki et al., 2007) and eventually of two ubiquitin-like conjugation systems.

The ubiquitin-like molecule Atg12 (ATG12 in mammals) is conjugated to Atg5 (ATG5) by Atg7 (ATG7) and Atg10 (ATG10), which act as E1 and E2 enzymes respectively. The Atg12-Atg5 conjugate forms a complex with Atg16 (Atg16L1) and acts as E3 enzyme for the conjugation of the ubiquitin-like molecule Atg8 to the head group of phosphatidylethanolamine (PE), a reaction known as Atg8 lipidation. In this reaction Atg7 and Atg3 (ATG3) act as E1 and E2 respectively (Klionsky and Schulman, 2014). Seven Atg8 homologs were identified in mammalian cells: LC3A, LC3B, LC3B2, LC3C, GABARAP, GABARAPL1 and GABARAPL2/GATE-16 (Tanida et al., 2004). Hereafter Atg8 and its homologs will be referred to as Atg8-family proteins. In order to allow conjugation to PE, Atg8-family proteins need to be activated by the cysteine protease Atg4 (ATG4A-D) (Hemelaar et al., 2003, Kirisako et al., 2000). The same protease is also responsible for the removal of Atg8-family proteins from the outer autophagosomal membrane upon autophagosome maturation.

Given the important role of ATG proteins in autophagy, much work in the past has focused on the characterization of this protein machinery both *in vivo* and *in vitro*. In order to fully understand the molecular mechanism of autophagosome formation, it is important to consider that the protein machinery is intimately linked to the membrane. With the development of assays that allowed the *in vitro* reconstitution of several aspects of autophagosome formation, using synthetic vesicles or cell fractions, reconstitution studies on autophagy have bloomed and contributed important insights in the molecular mechanism of autophagosome formation.

In this review we describe the main membrane modeling events taking place during autophagy, with particular attention to the reconstitution experiments employed to elucidate how the cellular machinery mediates them. This will underscore the advantages of reducing a process to its minimal components in order to understand the mechanisms by which these components act. We also discuss some of the pitfalls of current reconstitution experiments.

## 2 Origin of the isolation membrane

Although numerous cell compartments have been proposed as sources for autophagosomal membrane, its origin is still unclear (Lamb et al., 2013). Upon starvation, PI3P enriched sites on the ER recruit the Double FYVE Containing Protein 1 (DFCP1) and generate a ring-like structure named the omegasome, from which the isolation membrane, positive for Atg8-family proteins, emerges (Axe et al., 2008). Later it was shown by EM tomography that the isolation membrane grows out from the endoplasmic reticulum (ER) and that a physical connection exists between the ER and the isolation membrane (Hayashi-Nishino et al., 2009, Yla-Anttila et al., 2009). Consistent with an involvement of the ER, targeting of PI3K to the ER by ATG14L has been shown to be necessary for autophagy in mammalian cells (Hamasaki et al., 2013, Matsunaga et al., 2010). It was also suggested that the outer mitochondrial membrane contributes lipids to the autophagosome (Hailey et al., 2010) and that ATG14L is recruited to ER-mitochondria contact sites upon autophagy induction (Hamasaki et al., 2013). Nevertheless, the mechanisms that determine the precise place of autophagosome formation upon induction of autophagy in yeast and mammals are not well understood. Other compartments including the Golgi, the plasma membrane, ER exit sites (ERES) and endosomes have been reported to supply membranes for autophagosome formation and expansion (Graef et al., 2013, Longatti and Tooze, 2012, Ravikumar et al., 2010, van der Vaart et al., 2010).

Recently, it was reported that cell fractions enriched in the ER-Golgi Intermediate Compartment (ERGIC) were most potent in supporting LC3B lipidation *in vitro* (Ge et al., 2013). In particular, different membrane compartments were biochemically isolated from cells lacking ATG5, and thus LC3B lipidation activity. Subsequently, cytosol from starved wild type cells was added to these membrane fractions to reconstitute LC3B lipidation, which, in turn, served as marker for the functionality of the autophagic machinery. Consistent with the results from this semi *in vitro* assay, it was further demonstrated that a functional ERGIC is required *in vivo* for LC3B lipidation and that the ERGIC is enriched in the PI3K subunit ATG14L. In a follow up study it was demonstrated that, upon starvation, PI3K activation leads to the recruitment of COPII proteins to the ERGIC, causing the budding of small vesicles that are active in LC3B lipidation (Ge et al., 2014a). Based on these results, it was suggested that these vesicles are a possible source of autophagosomal membrane.

Although this study does not exclude the contribution of other membrane compartments to the autophagosome, it strongly points at the ERGIC as an important source of vesicles for the early stages of isolation membrane nucleation in mammalian cells. This work is relevant also from the technical point of view. In fact, it represents the first step towards the isolation of autophagic membrane precursors, which could lead to a better understanding of their lipid composition in the future. In *S. cerevisiae*, where the ERGIC has not been found (Appenzeller-Herzog and Hauri, 2006), COPII vesicles generated from the ERES might similarly contribute to generate autophagic membranes for PAS assembly and isolation membrane growth. In fact, the edges of the isolation membrane were shown to be in close proximity to the ERES (Graef et al., 2013, Suzuki et al., 2013).

## 3 PAS organization

In yeast the PAS is a perivacuolar site to which all the core autophagic machinery localizes (Suzuki et al., 2001). Therefore, it is generally assumed that it is the site of autophagosome formation. Among the proteins recruited to the PAS is the transmembrane protein Atg9. It was calculated that an average of three Atg9-containing vesicles are needed to nucleate the isolation membrane (Yamamoto et al., 2012) (Fig. 1A). However, it is not clear how Atg9 vesicles are recruited, organized and remodeled.

Upon starvation Atg17, in complex with Atg29 and Atg31, is one of the first proteins recruited to the PAS and it is responsible for PAS organization (Kawamata et al., 2008). Recently, the crystal structure of Atg17-Atg29-Atg31 complex was solved and it revealed that Atg17 dimerizes, adopting a double crescent conformation, with each Atg17 monomer resembling the characteristic shape of membrane binding BAR domain proteins (Ragusa et al., 2012). However, unlike classical BAR domains, Atg17, at least when in complex with Atg29 and Atg31, did not show any detectable binding to small unilamellar vesicles (SUVs). Rather, Atg17 is targeted to the membrane via its interaction with the EAT (Early Autophagy Targeting/Tethering) domain of Atg1, which was shown to selectively bind to highly curved vesicles *in vitro*. On the basis of these results, the authors suggested that Atg17, in complex with Atg1, could behave as a membrane curvature sensor and cluster Atg9 vesicles. In particular, it was proposed that the Atg1 EAT domain recruits and tethers vesicles, while the Atg17-Atg29-Atg31 complex acts as a scaffold for the spatial organization of these vesicles at the PAS. They also proposed a regulatory mechanism in which Atg29-Atg31 dimer blocks the vesicle binding surface to prevent unspecific vesicle tethering and enable selection for Atg9 vesicles.

In a more recent study the potential mechanism of Atg9 vesicle tethering by the Atg1 complex was recapitulated *in vitro* (Rao et al., 2016). The study showed that the recombinant *S. cerevisiae* Atg1 pentameric complex, composed of Atg1, Atg13, Atg17, Atg29 and Atg31, is able to bind and tether proteoliposomes containing a truncated form of Atg9. Atg17 was shown to directly interact with Atg9 transmembrane core, while Atg29-Atg31 dimer competes with Atg9 for Atg17 binding. It was further demonstrated that binding of Atg1-Atg13 dimer to Atg17-Atg29-Atg31 trimer, activates Atg17 and promotes its binding to Atg9 proteoliposomes. Since Atg13 was shown to interact with Atg17 (Fujioka et al., 2014), they suggested that binding of Atg1-Atg13 dimer to Atg17-Atg29-Atg31 complex induces a conformational change in the trimer, which leads to the displacement of Atg29-Atg31 from the Atg9 binding site on Atg17. The detailed mechanism of this displacement remains however unclear and may only be revealed by high resolution structures. Based on these data it was proposed that Atg9 vesicle tethering is mediated by the interaction of Atg17 with the Atg9 transmembrane core rather than via the interaction of the Atg1 EAT domain with membranes (Ragusa et al., 2012, Rao et al., 2016).

The early stages of autophagosome nucleation, described in this section, take place upon autophagy induction by starvation and upstream of Atg8 lipidation. It is possible that Atg9 vesicles form the initial scaffold for the recruitment of the ATG machinery, which in turn is responsible for the conjugation of Atg8-family proteins to PE on the ERGIC/ERES generated COPII vesicles (Fig. 1A). We refer the readers to a recent review by Ge et al. where an interesting model is presented in which the TRAPPIII complex mediates heterotypic tethering of Atg9 and COPII vesicles (Ge et al., 2014b). Downstream of the vesicles tethering events, SNARE proteins (Soluble NSF Attachment protein REceptor) may play an important role in vesicles fusion (Nair et al., 2011). Very little is known about isolation membrane nucleation in mammalian cells. Since autophagy can be initiated at different sites in the cell and in response to many different stimuli, a more complex regulation is likely. Recently, the structure of human ATG13-ATG101 HORMA domains was solved, suggesting that ULK1 complex could behave as a hub for the interaction with different, yet unknown, regulatory proteins (Qi et al., 2015, Suzuki et al., 2015a).

## 4 Isolation membrane expansion

Atg8-family proteins are responsible for isolation membrane growth and autophagosome formation. It was originally shown that the amount of Atg8 in the cell correlates with the size of the autophagosomes, without affecting their number (Xie et al., 2008). Moreover, autophagosome formation is strongly compromised when Atg8-family protein lipidation is abolished (Kishi-Itakura et al., 2014, Komatsu et al., 2005, Sou et al., 2008). Nonetheless, the formation of isolation membranes is still observed in cells where Atg8-family protein lipidation is impaired by ATG5 or ATG3 deletion (Kishi-Itakura et al., 2014, Mizushima et al., 2001, Sou et al., 2008). Taken together these data suggest that the isolation membrane is nucleated upon autophagy induction by starvation in an Atg8 independent manner. Subsequently, the recruitment of the conjugation machinery to the newly formed isolation membrane, leads to Atg8-family protein lipidation and promotion of isolation membrane expansion and closure by a mechanism that is still unclear. The widely proposed model is that isolation membranes are generated by fusion of small vesicles to the growing isolation membrane. On the basis of *in vitro* experiments, it was suggested that Atg8 could aid this mechanism by promoting vesicles tethering and hemifusion (Nakatogawa et al., 2007). In addition, the human Atg8 homologues LC3B and GATE-16 and the *C. elegans* Atg8 homologs LGG-1 and LGG-2 mediate homotypic fusion of SUVs *in vitro* (Weidberg et al., 2011, Wu et al., 2015). In all these studies the N-terminal residues of the proteins were proposed to be responsible for the fusion event thus important for autophagosome formation *in vivo*. The significance of the fusogenic activity of Atg8-family proteins *in vivo* was questioned (Nair et al., 2011) when it was shown that yeast Atg8 and human LC3B do not promote vesicles fusion when physiological concentrations of PE (below 30 mol%) are used in the liposomes. However, other studies showed that LC3B, LGG1 and LGG2 mediate liposomes fusion with PE concentrations as low as 20 mol% (Weidberg et al., 2011, Wu et al., 2015). The discrepancy between those results may be explained by different amounts of unsaturated lipids used in the studies. The membrane remodeling ability of Atg8 was further investigated by reconstituting the lipidation reaction on GUVs. GUVs that harbor Atg8 show massive tethering but no obvious fusion (Knorr et al., 2014, Romanov et al., 2012). Tethering of GUVs was observed only above a certain threshold of Atg8 concentration on the membrane implying that the tethering activity of individual Atg8 proteins is low (Knorr et al., 2014). In the same study it was also shown that at high PE concentrations Atg8 has a slight preference for highly curved membranes and that it can induce deformation of GUVs (Knorr et al., 2014).

It was recently shown that the fusogenic ability of Atg8-family proteins correlates with the amount of packing defects on the membranes and their ability to promote hemifusion intermediates (Landajuela et al., 2016). Introduction of cone-shaped lipids like diacylglycerol (DAG) or cardiolipin (CL) in SUVs stimulates GABARAP/GATE-16-mediated membrane fusion. On the contrary, when inverted cone-shaped lipids, like lyso-phosphatidylcholine (LPC), were used, lipid mixing was inhibited. Furthermore, the level of lipid mixing was shown to be inversely proportional to the vesicles radius. This effect was independent from the efficiency of the lipidation reaction, since Atg8-family proteins were directly cross-linked to PE. Taken together, those results suggest, analogous to many other membrane fusion events, a correlation between Atg8-family protein-mediated membrane fusion and the membrane composition.

Given the direct physical connection between the isolation membrane and the ER, isolation membrane expansion could be achieved by lipid transfer from the ER. This potential mechanism would generate isolation membranes with a lipid composition similar to the ER, unless integral membrane proteins are present that regulate lipid transfer between the two compartments. Another interesting possibility is that local lipid synthesis or transport by transfer proteins could aid isolation membrane growth and shaping, although no direct link between the lipid biosynthesis and the autophagic machinery was reported. Further studies in this direction could lead to a better understanding of how the lipid composition of the isolation membrane is achieved and allow their faithful reconstitution *in vitro*.

## 5 Autophagosome shaping

**Fig. 2:**
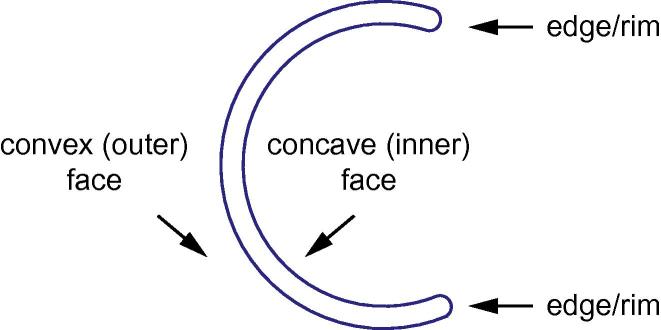
Topology of the isolation membrane. The highly curved edges, the convex (outer) and concave (inner) face are shown.

Electron microscopy pictures have shown that in the early stages of autophagosome formation the isolation membrane appears as a cup-shaped double membrane with highly curved edges (Hayashi-Nishino et al., 2009, Yla-Anttila et al., 2009) (Fig. 2). This topology is not energetically favorable since the lipids in the highly curved edges are under curvature stress (Knorr et al., 2012). How this structure is stabilized is unclear since scaffolds that would act like a coat, similarly to clathrin or COPs, were never observed on isolation membranes in cells. Perhaps, the ATG protein machinery itself contributes to the creation and stabilization of the isolation membrane shape.

PI3P is present in isolation membranes and upon induction of autophagosome formation by starvation is needed to recruit downstream ATG machinery including WD40 repeat proteins (Baskaran et al., 2012, Krick et al., 2012). The PI3Kc1 complex that generates the PI3P is itself a large protein complex that may, apart from its enzymatic activity, help to shape and organize the isolation membrane (Baskaran et al., 2014, Fan et al., 2011, Rostislavleva et al., 2015).

ATG3, the E2-like enzyme involved in the conjugation of Atg8-family proteins to PE has a N-terminal amphipathic helix that binds selectively to highly curved membranes. The affinity of ATG3 for liposomes of different size and composition was extensively tested *in vitro*, and mutations altering the distribution of hydrophobic amino acids on the helix have a direct effect on LC3B/GABARAPL1-2 lipidation *in vivo* (Nath et al., 2014). This led the authors to place ATG3, and consequently the Atg8-family proteins conjugation reaction, at the highly curved rim of the growing isolation membrane. Furthermore, it was shown that also the Atg12-Atg5-Atg16 complex can be recruited to membranes through a membrane binding site in Atg5 (Romanov et al., 2012). Since Atg5 showed a preference for liposomes containing conical-shaped lipids, the complex might select membranes bearing packing defects, like the isolation membrane edge. It is also conceivable that the Atg8-family proteins conjugation machinery associates with the growing isolation membrane preferentially at the edge, but eventually diffuses along the membrane.

How the shape and size of the autophagosome as a whole is generated is unclear. During cargo-induced selective autophagy, bulky cargo material may act as scaffold (Zaffagnini and Martens, 2016). Indeed, it was shown that the interaction of cargo receptors with membrane localized Atg8-family proteins results in close apposition of the membrane and the cargo and thereby in membrane bending (Sawa-Makarska and Martens, 2014, Sawa-Makarska et al., 2014, Wurzer et al., 2015). In particular, a series of *in vitro* reconstitution experiments using cargo-mimetic beads and Giant Unilamellar Vesicles (GUVs) showed that oligomerization of the cargo receptor p62, or multiple LIR motifs on the cargo receptor Atg19 bind concentrated Atg8-family proteins with high avidity and shape the membrane using the cargo as template (Sawa-Makarska et al., 2014, Wurzer et al., 2015). This mechanism of membrane shaping, employed when bulky cargo material is present, is likely to act in conjunction with other mechanisms that are employed during starvation induced autophagy and which are still enigmatic.

Atg8-family proteins decorate both the convex and the concave surfaces (Fig. 2) of the forming autophagosome. A reconstitution study addressed the autophagosome shaping role of Atg8 on the convex face of the isolation membrane (Kaufmann et al., 2014). It was shown that Atg8 recruits the Atg12-Atg5-Atg16 complex to GUVs. In turn, oligomerization of Atg16 through its coiled-coil domain contributes to the creation a two-dimensional scaffold that immobilizes Atg8 conjugated on the membrane. It was proposed that this protein scaffold stabilizes the isolation membrane while allowing a certain degree of plasticity to adapt to the size and shape of different cargo particles (Fig. 1B). It was additionally shown that the cargo receptor Atg32 competes with the Atg12-Atg5-Atg16 complex for Atg8 binding, suggesting that this difference in binding affinity explains the exclusive localization of Atg12-Atg5-Atg16 on the convex (outer) face of the isolation membrane, while the cargo is located on the concave (inner) face. It remains to be confirmed that a scaffold exists on isolation membranes in cells. In fact, functional impairment of Atg8-family protein lipidation in mammalian cells by ATG5 deletion, leads to isolation membranes which conserve their typical shape, but fail to close, arguing that Atg8-family protein lipidation and the ATG12-ATG5-ATG16L complex are not essential for isolation membrane shaping and that other mechanism may (co)-exist in mammalian cells (Kishi-Itakura et al., 2014). Recently, the actin cytoskeleton nucleated by CapZ was implicated in the shaping of isolation membranes by acting similarly to bulky cargo during cargo-induced autophagy (Mi et al., 2015) (Fig. 1B). It is possible that multiple mechanisms, all acting in the same direction, mediate robust shaping of the autophagosomal membrane. This would make the reconstitution of the process from purified components very challenging. It is also conceivable that the growing isolation membrane assumes a round shape because it represents the thermodynamically most favorable state, given the energy cost to maintain the highly curved edges of the isolation membrane (Knorr et al., 2012).

## 6 Isolation membrane closure and fusion of autophagosomes with the lysosome

A mature autophagosome is generated when the isolation membrane closes. It represents the transition from a single membrane structure to a double membrane vesicle within which a portion of the cytoplasm is trapped (Fig. 1B). The mechanism of isolation membrane closure is still obscure.

It was shown that Atg8-family proteins are important for isolation membrane closure in mammalian cells (Fujita et al., 2008, Kishi-Itakura et al., 2014), but their mechanism of action is still unclear. Given their fusogenic activity it is possible that they directly mediate the merger of the membranes (see discussion above). On the other hand, isolation membrane closure may actually be a fission (or scission) event, rather than fusion and therefore similar to the remodeling event characterizing cytokinesis or Multi Vesicular Body (MVB) formation. The typical machinery mediating fission events of this topology in the cells is the ESCRT (Endosomal Sorting Complex Required for Transfer) machinery (Hurley, 2015, Rusten et al., 2012).

Some groups have reported that ESCRT complexes are involved in autophagy and more precisely in isolation membrane closure (Lee et al., 2007, Rusten et al., 2007, Spitzer et al., 2015). Indeed, it would not be surprising if the ESCRT machinery would mediate autophagosome closure, but it is challenging to separate direct from indirect effects upon interference with ESCRTs function *in vivo*, as these proteins are required for a functional endo-lysosomal system. Reconstitution experiments could potentially show if ESCRTs could, at least in principle, mediate isolation membrane closure, but so far it has been impossible to produce open isolation membranes/vesicles *in vitro*.

Mature autophagosomes fuse with the vacuole/lysosome. Upon fusion, the inner autophagosomal membrane and all its content are degraded (Fig. 1B). It is essential that an autophagosome is mature (closed) before the fusion with the lysosome occurs, since otherwise the intermembrane content, rather than the actual cargo, would be delivered into the lysosomal lumen. It is thought that the removal of Atg8-family proteins from the outer autophagosomal membrane, mediated by Atg4, could represent a signal for the recruitment of the fusion machinery. In this case, what triggers Atg8-family proteins cleavage from the outer autophagosomal membrane? It is possible that Atg8-family protein lipidation and cleavage occur simultaneously during autophagosome formation (Noda et al., 2009). If the lipidation reaction proceeds with a higher rate, the overall outcome is a Atg8-family proteins decorated isolation membrane. Upon autophagosome closure, the highly curved edge of the isolation membrane, where the lipidation machinery was proposed to be recruited (Nath et al., 2014), ceases to exist. This would cause an arrest of the lipidation reaction, while Atg4 activity would prevail.

PI3P hydrolysis is required for dissociation of ATG proteins, including Atg8, from the autophagosome (Cebollero et al., 2012), but it is unclear how PI3P hydrolysis and isolation membrane closure are coupled. Perhaps the PI3K and the PI3P phosphatase activities are spatially separated such that the kinase activity localizes to the concave side and the phosphatase activity localizes to the convex side. After isolation membrane closure, the PI3P generated at the concave (inner) side would not be able to diffuse to the convex (outer) side, resulting in the depletion of PI3P from the outer membrane, which in turn may lead to Atg8 cleavage from the outer membrane. For this mechanism to occur during autophagy, the PI3K needs to be localized inside the mature autophagosome, which is difficult to envision in cases where the autophagosome contains bulky cargo material such as mitochondria, pathogens or prApe1 complexes. It is likely that multiple mechanisms act in concert to robustly couple isolation membrane closure to Atg8-family protein de-lipidation.

In yeast, autophagosomes are generated close to the vacuole and therefore do not require long-range transport before fusion. In mammalian cells, however, autophagosomes are formed at multiple sites and must be transported to lysosomes before fusion. Many of the adaptors required for transport and fusion are recruited to the autophagosomal membrane via their interaction with Atg8-family proteins (for review see Stolz et al. (2014)). In particular, LC3B molecules conjugated to the outer autophagosomal membrane are needed for the movement of the complete autophagosome along the microtubules, towards the lysosome (Fu et al., 2014, Kimura et al., 2008). If LC3B is cleaved from the mature autophagosome too early, transport to the lysosome might be impaired. An intriguing possibility is the existence of a non-cleavable sub-population of LC3B on the isolation membrane. *In vitro* reconstitution of LC3B lipidation reaction on synthetic liposomes showed that in addition to PE, the conjugation machinery can target also phosphatidylserine (PS) (Sou et al., 2006). It remains to be established if LC3B-PS is generated also *in vivo*, or if it is a non-specific product of the *in vitro* reaction. Nevertheless, if LC3B-PS existed in the cell, it would not be a substrate of ATG4 and hence could not be cleaved (Sou et al., 2006). Perhaps a small, difficult to detect pool of LC3B-PS exists that serves to recruit adaptors to the completed autophagosome. In summary, the precise signals that trigger autophagosome maturation and fusion with the lysosome remain a fascinating topic for future studies.

The machinery involved in autophagosome-lysosome fusion includes Rab proteins, HOPS and SNAREs and it was comprehensively reviewed recently (Ganley, 2013). *In vitro* reconstitution experiments were used to characterize this final step of autophagy. They showed that the SNARE complex composed of VAMP8-STX17-SNAP29 promotes fusion of membrane vesicles *in vitro* (Diao et al., 2015). Additionally, ATG14L can stabilize the SNARE complex and enhance its membrane fusion activity *in vitro*. It was also shown that ATG14L alone can mediate liposome tethering and aid SNARE-mediated membrane fusion.

## 7 Discussion and future perspective

In the past few years, *in vitro* reconstitutions, often coupled with light microscopy or electron microscopy, have provided important contributions to the study of autophagy. In particular, reconstitution experiments enable one to determine which factors are not only necessary but also sufficient for a given process.

The initial observations of autophagosome formation in cells and tissues were based on morphological studies employing electron microscopy (Arstila and Trump, 1968, De Duve and Wattiaux, 1966, Mortimore and Schworer, 1977, Schworer and Mortimore, 1979). After autophagy proteins were identified by genetics, their behavior could be followed by fluorescence microscopy (Harding et al., 1995, Tsukada and Ohsumi, 1993). Later, *in vitro* reconstituted systems were used to characterize the ATG machinery and understand the molecular mechanism of autophagosome formation. As described above, reconstitution experiments were used in attempts to understand and recapitulate almost every step of autophagosome formation. Synthetic vesicles (SUVs, LUVs, GUVs) offered the possibility to directly address the membrane binding ability of several ATG proteins (Kaufmann et al., 2014, Krick et al., 2012, Nakatogawa et al., 2007, Nath et al., 2014, Ragusa et al., 2012, Rao et al., 2016, Romanov et al., 2012). *In vitro* assays using SUVs were devised to measure protein-mediated membrane tethering and fusion, while GUVs have been used to investigate the membrane deformation/modeling ability of autophagy related proteins (Kaufmann et al., 2014, Knorr et al., 2014, Sawa-Makarska et al., 2014, Wurzer et al., 2015).

While reconstitution experiments have provided unique mechanistic insights into the action of the ATG machinery, it is conspicuous that many ATG proteins were reported to bind highly curved vesicles and to tether membranes. The reason may be that these parameters are relatively easy to detect in the *in vitro* systems currently employed, and therefore some degree of caution should be applied. In many cases the proteins showed a strong preference for small vesicles, with a dimeter below 50 nm, relatively independently of their lipid composition. Packing of biological membranes is influenced by their lipid composition: while cylinder-shaped lipids give rise to tightly packed bilayers, introduction of cone-shaped lipids results in packing defects and membrane stress (Vamparys et al., 2013). The overall shape of a lipid is influenced by the size of the polar head group and by the degree of saturation of the acyl-chains. As a result, the final lipid mixture used to generate liposomes directly influences the lipid packing. Moreover, the spontaneous radius of curvature of any membrane is dictated by the relative amounts of conical and cylindrical lipids. Liposome formation by sonication induces high membrane curvature, which deviates greatly from the spontaneous curvature radius, thus packing defects are created. For ALPS motifs containing protein it was shown that exposed hydrophobic residues efficiently absorb into a lipid bilayer, (Antonny, 2011) and their membrane binding ability dramatically increases upon introduction of membrane packing defects (Bigay et al., 2003, Drin et al., 2007). However, if the degree of packing defects exceeds a certain limit and especially in combination with a high abundance of charged lipids, some proteins may show membrane binding, although the lipid bilayer is not a natural binding partner. Additionally, protein-membrane interactions are often characterized by a low binding affinity and *in vivo* specificity is achieved by co-incidence of multiple weak interactions. Thus, if a protein has two or more weak membrane binding sites and the additional binding partners are missing, this will result in high off rates of the protein-membrane interaction. This in turn will facilitate membrane tethering in *in vitro* systems, where vesicles and proteins are used at high concentrations. Furthermore, tethering, as opposed to other membrane directed events, is relatively easy to detect by light scattering or microscopy and may therefore often be over interpreted.

In addition, the head groups of lipids have critical roles in the recruitment of proteins to membranes. While phosphoinositides such as PI3P are relatively well studied, other lipids such as phosphatidic acid may also have important roles during autophagosome formation (Dall’Armi et al., 2013) and their inclusion in reconstitution experiments may be important to recapitulate certain aspects of this process.

SUVs have been used to determine the ability of some ATG proteins to mediate membrane fusion or hemifusion, as assessed by lipid mixing assay (Struck et al., 1981). In some studies, membrane fusion was analyzed with liposomes containing high, perhaps non-physiological concentration of PE (above 40 mol%). PE is an intrinsically fusogenic lipid and, if used at high concentration, can promote membrane fusion, independently of any protein (Chernomordik and Kozlov, 2003). Similar considerations apply to the study of SNARE-mediated membrane fusion. SNARE complex assembly is slow and rate limiting in *in vitro* fusion experiments (Jahn and Scheller, 2006). Thus, many factors that tether vesicles will aid SNARE complex assembly by bringing the trans-SNAREs into proximity, which in turn, will stimulate fusion.

GUVs and lipid bilayers are excellent tools to study protein-mediated membrane deformation events and they are widely used in autophagy reconstitution experiments, However, since they approximate a flat surface, they are not perfectly suited to study events taking place in proximity of the highly curved edges of the isolation membrane. Also, GUVs can be very flexible and prone to deformation, depending on the membrane and buffer composition. Thus, it is important to back up reconstitution approaches with additional experiments.

The following advances will greatly facilitate the successful reconstitution of major steps during autophagy. First, a better ultra-structural description of the process, similar to what has been obtained for endocytosis (Kukulski et al., 2012), will provide insights into the actual steps occurring during autophagosome formation. Second, a thorough characterization of the lipid composition of the autophagosomal membrane and its precursors, similar to what has been obtained for synaptic vesicles (Takamori et al., 2006), will help to choose the correct lipid composition of the vesicles used in the *in vitro* systems. Membrane fractionation techniques coupled with affinity purification of membrane binding proteins involved in autophagy could lead to the isolation of autophagic membranes suitable for lipidomic analysis. Further, more sophisticated methods that recapitulate the shape of the growing isolation membrane, i.e. two flexible cup-shaped membranes separated by a small gap and connected by highly curved edges, are needed. Micromanipulation of GUVs was used to obtain tubules with a controllable radius (Knorr et al., 2014), but so far no other shapes could be made. Nanoparticle science might be employed in the future to obtain cup-shaped isolation membranes. Synthetic nanomaterials are extremely versatile in that they can be produced in any size and shape. Recently, biopolymers have been successfully coated with cellular membranes to facilitate their delivery in the human body (Hu et al., 2011). A cup-shaped biopolymer coated with synthetic or purified autophagic membranes, could mimic isolation membranes at very early stages. This tool could be used to study accurately the events taking place at the rim of the isolation membrane or on its concave and convex faces. Furthermore, it might be employed as seed to investigate the mechanism of autophagosome growth and shaping by supplying a combination of vesicles and recombinant proteins or cell lysates. The reconstitution of the connections between the isolation membrane and the ER might be even more complex. Perhaps this is best tackled using selective autophagy as model. A bulky model cargo could be brought in proximity to GUVs to initiate isolation membrane formation. The GUVs could be manipulated such that they contain the right lipids and transmembrane proteins.

It is likely that a combination of approaches will be required to obtain insights into the inner working of the fascinating autophagic machinery and to finally achieve a full reconstitution of all steps of this unique biological process.
